# P-2224. Clinical and Public Health Implications of Tularemia Diagnoses by Microbial Cell-Free DNA Testing

**DOI:** 10.1093/ofid/ofae631.2378

**Published:** 2025-01-29

**Authors:** Grace E Marx, Elizabeth A Dietrich, Laurel Respicio-Kingry, Shannan Rich, Sharjeel Ahmad, Nitin Das KP, Elio Jabra, Mobeen Haider, Amir Khan, Constance C Austin, Ahmad AlSalman, Julie Coughlin, Markus Plate, Joel Ackelsberg, Jennifer White, Thara Damodaran, Matthew Nichols, Benjamin McMillion, Allan M Seibert, Hannah Rettler, Jeannine Petersen, Sarah Y Park

**Affiliations:** Centers for Disease Control and Prevention, Fort Collins, Colorado; Centers for Disease Control and Prevention, Fort Collins, Colorado; Centers for Disease Control and Prevention, Fort Collins, Colorado; Centers for Disease Control and Prevention, Fort Collins, Colorado; University of Illinois College of Medicine, Peoria, IL; UIC; Carle Foundation Hospital, Urbana, Illinois; Carle Foundation Hospital, Urbana, Illinois; Carle Foundation Hospital, Urbana, Illinois; Illinois Department of Public Health, Springfield, Illinois; UnityPoint Health, Des Moines, Iowa; Iowa Department of Health and Human Services, Des Moines, Iowa; Weill Cornell Medical College/ New York Presbyterian Hospital , New York; NYC Department of Health and Mental Hygiene, New York, New York; New York State Department of Health, Albany, New York; Mercy Hospital Oklahoma City, Oklahoma City, Oklahoma; Oklahoma State Department of Health, Oklahoma City, Oklahoma; University of Utah School of Medicine, Salt Lake City, UT; Intermountain Health, Murray, UT; Utah Department of Health and Human Services, Salt Lake City, Utah; Centers for Disease Control and Prevention, Fort Collins, Colorado; Karius, Inc., Redwood City, California

## Abstract

**Background:**

Tularemia is a nationally notifiable disease and Category A potential bioterrorist threat caused by *Francisella tularensis*. Microbial cell-free DNA (mcfDNA) sequencing is a pathogen-agnostic diagnostic method using human plasma. mcfDNA detections of *F. tularensis* are not currently included in public health surveillance criteria for a tularemia case and are not automatically reported to public health jurisdictions. We reviewed recent *F. tularensis* mcfDNA detections to better understand their clinical and public health implications.

**Table 1.**
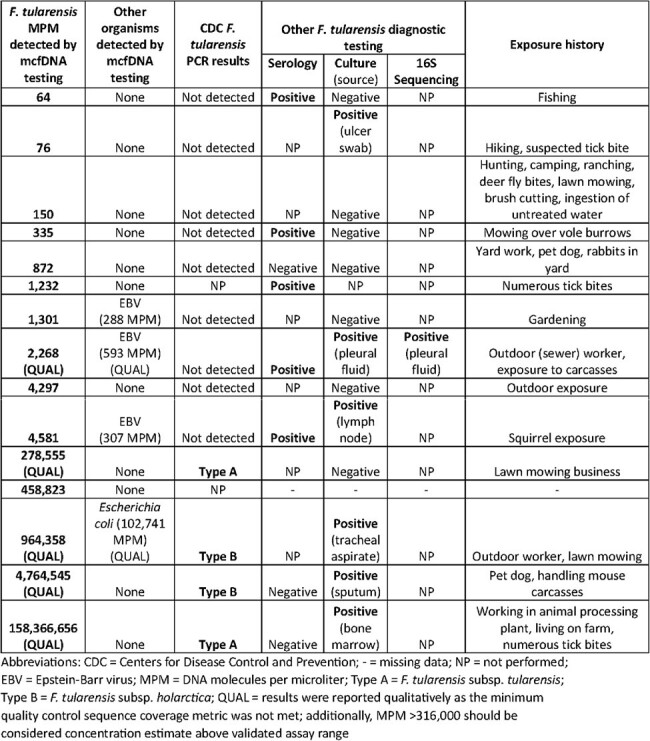
Francisella tularensis diagnostic test results and reported exposure histories among 15 patients with F. tularensis detected by mcfDNA sequencing — United States, 2018–2023.

**Methods:**

Available residual patient samples, in which *F. tularensis* mcfDNA were detected by the Karius CLIA certified/CAP accredited laboratory, were sent to the Centers for Disease Control and Prevention (CDC) for *F. tularensis* testing by PCR. Treating clinicians and local/state public health jurisdictions where these patients lived at the time of testing were invited to complete a survey regarding clinical details and public health reporting.

**Table 2.**
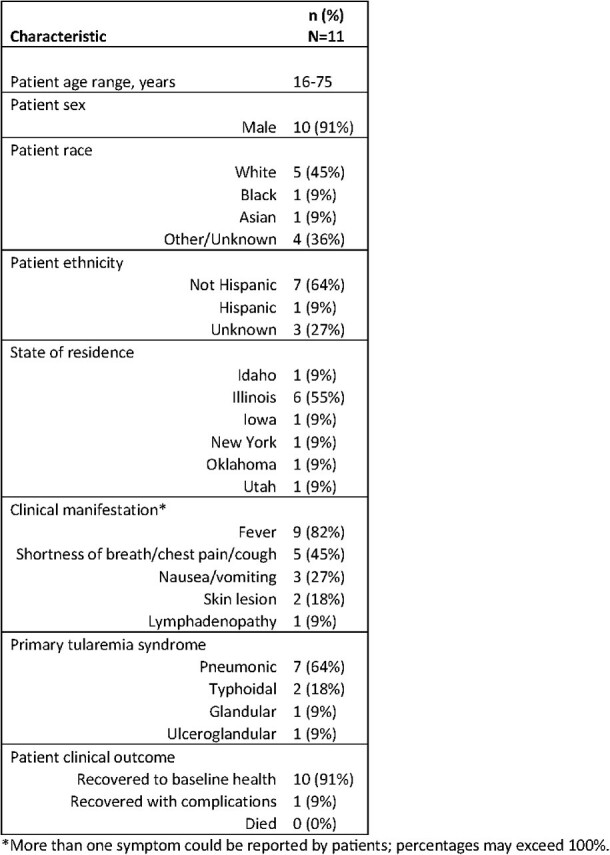
Demographic and clinical characteristics of patients with Francisella tularensis detected by microbial cell-free DNA (mcfDNA) testing, as reported by the ordering clinician — United States, 2018–2023.

**Results:**

During 2018–2023, mcfDNA sequencing detected *F. tularensis* in 15 patient samples in 6 states. CDC detected *F. tularensis* by PCR in 4 (31%) of 13 residual samples, all in samples with higher DNA quantities. All invited jurisdictions (n=6) and most clinicians (8/13, 62%) participated in the survey. Most cases (12/15, 80%) were reported to public health; 3 of 12 (25%) were determined not to meet the tularemia surveillance case definition, and only 1 case reported to CDC mentioned mcfDNA results. Among 11 cases with clinical information, tularemia was not suspected before receipt of mcfDNA results in 10 (91%), despite all patients reporting outdoor activities that could increase *F. tularensis* exposure risk. Other diagnostic tests for tularemia were positive in 7 of 11 (64%) cases; blood cultures were negative in all cases. mcfDNA results changed clinical management in all cases, resulting in antibiotic changes.

**Figure 1.**
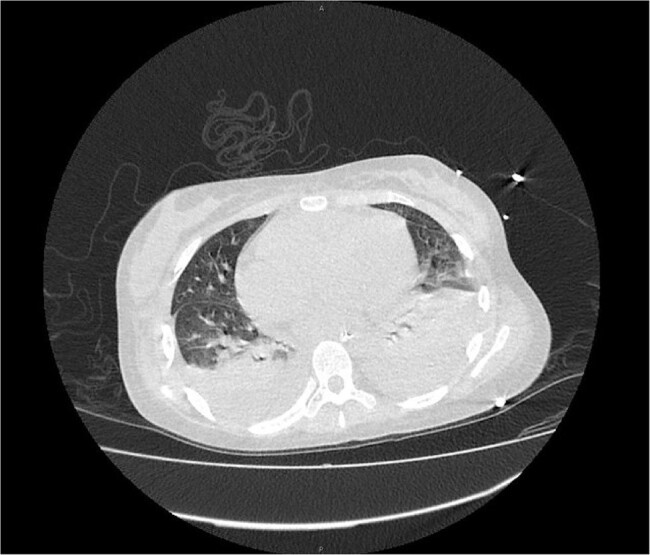
Cross-sectional computed tomography of the lungs in a patient diagnosed with pneumonic tularemia by microbial cell-free DNA (mcfDNA) testing, showing bilateral moderate pleural effusion and right upper and middle lobe pulmonary nodules. This patient also exhibited nonspecific mediastinal and bilateral axillary lymphadenopathy and typhoidal symptoms. In this case, mcfDNA testing identified Francisella tularensis nearly 50 hours before any conventional testing confirmed the diagnosis and prompted changes in antibiotic therapy and infection prevention efforts to begin.

**Conclusion:**

mcfDNA appears to be clinically impactful and highly sensitive for diagnosis of tularemia, particularly in febrile patients with consistent exposure history. New diagnostic methods may pose a challenge to public health surveillance and require changes to case definitions and reporting practices to ensure robustness of public health utility and timely prevention efforts.

**Figure 2.**
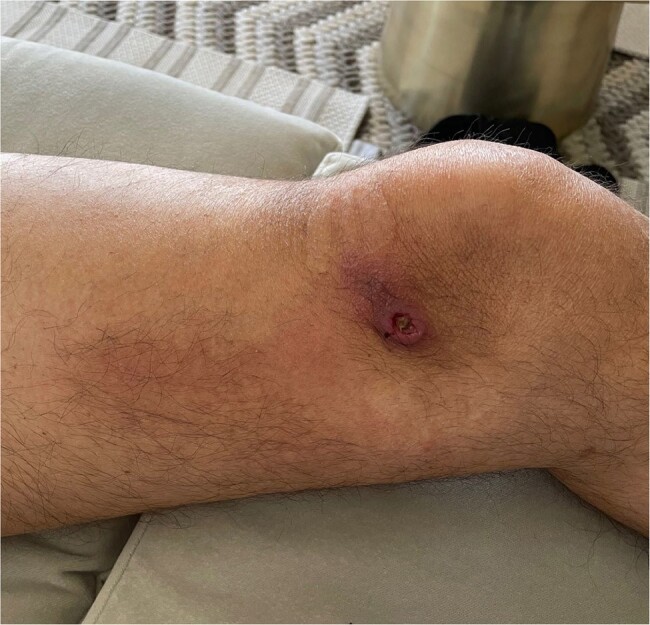
Left medial knee with 3 cm nodular ulcer with purulence and adjacent erythema at location of suspected tick bite in a patient diagnosed with ulceroglandular tularemia by microbial cell-free DNA (mcfDNA) testing.

**Disclosures:**

Sarah Y. Park, MD, FAAP, Karius, Inc.: employee

